# Drug Discovery for *Mycobacterium tuberculosis* Using Structure-Based Computer-Aided Drug Design Approach

**DOI:** 10.3390/ijms222413259

**Published:** 2021-12-09

**Authors:** Murtala A. Ejalonibu, Segun A. Ogundare, Ahmed A. Elrashedy, Morufat A. Ejalonibu, Monsurat M. Lawal, Ndumiso N. Mhlongo, Hezekiel M. Kumalo

**Affiliations:** 1 Biomolecular Modeling Research Unit, Discipline of Medical Biochemistry, School of Laboratory Medicine and Medical Science, University of KwaZulu-Natal, Durban 4001, South Africa; mejalonibu@yahoo.com (M.A.E.); almarufacrown@gmail.com (M.A.E.); mhlongon4@ukzn.ac.za (N.N.M.); 2 Department of Chemical Sciences, Olabisi Onabanjo University, Ago-Iwoye 120107, Nigeria; ajibola32@gmail.com; 3 Natural and Microbial Product Department, National Research Centre, Dokki 12622, Egypt; ahmedelrashedy45@gmail.com

**Keywords:** *Mycobacterium tuberculosis*, computational drug design, molecular docking, anti-tuberculosis, structure-based drug design

## Abstract

Developing new, more effective antibiotics against resistant *Mycobacterium tuberculosis* that inhibit its essential proteins is an appealing strategy for combating the global tuberculosis (TB) epidemic. Finding a compound that can target a particular cavity in a protein and interrupt its enzymatic activity is the crucial objective of drug design and discovery. Such a compound is then subjected to different tests, including clinical trials, to study its effectiveness against the pathogen in the host. In recent times, new techniques, which involve computational and analytical methods, enhanced the chances of drug development, as opposed to traditional drug design methods, which are laborious and time-consuming. The computational techniques in drug design have been improved with a new generation of software used to develop and optimize active compounds that can be used in future chemotherapeutic development to combat global tuberculosis resistance. This review provides an overview of the evolution of tuberculosis resistance, existing drug management, and the design of new anti-tuberculosis drugs developed based on the contributions of computational techniques. Also, we show an appraisal of available software and databases on computational drug design with an insight into the application of this software and databases in the development of anti-tubercular drugs. The review features a perspective involving machine learning, artificial intelligence, quantum computing, and CRISPR combination with available computational techniques as a prospective pathway to design new anti-tubercular drugs to combat resistant tuberculosis.

## 1. Introduction

Robert Koch identified the etiological agent of tuberculosis (TB) as *Mycobacterium tuberculosis* (Mtb) [1]. TB generates a lot of concerns as a contagious disease that poses a high risk to public health globally. Despite the available anti-tubercular drugs introduced over the years, TB remains one of the leading causes of death globally [2]. According to the World Health Organization (WHO), it is the most common infection caused by a single bacterium. About 10 million people were diagnosed with TB in 2017, and 558,000 of them showed resistance to the most effective first-line medication, rifampicin. According to another WHO survey, an estimated 1.5 million deaths occurred in 2018 [3]. It infects about a third of the world’s population and kills approximately 1.7–1.8 million people per year, demonstrating the failure to find new antibiotics to conquer this deadly disease [4]. Therefore, antimicrobial compounds that are effective against Mtb are desperately required to tackle this global epidemic, worsened by resistance to medication, long-time treatment schedule, and co-infection, especially with Human Immunodeficiency Virus (HIV). In more than 40 years, no new antibiotic to treat TB has been created [4,5].

Recently, phenotypic screening efforts using commercial vendor libraries evolved toward identifying compounds that inhibit Mtb development [6,7,8]. This intervention gives a ray of hope in the search for new therapeutics against Mtb. The urgency to end the Mtb epidemic requires improvement in diagnostic tools and the efficacy of therapeutics used in treating TB in diagnosed patients. This intervention reduces the treatment regimens usually required with strict compliance to ensure effective treatment. Rapid and cheap diagnostic test kits that can be readily accessible to the public aids early diagnosis, while drugs with multiple targets go a long way to improve the outcome of treatment [9]. There is urgent attention to deliver new potential active antimicrobial agents to scale down the resistant TB strains. Many strategies and efforts have been adopted, which involved the structure-based design of inhibitors for a single target pathogen through computational methods [10,11,12,13].

Target drug discovery begins with identifying and studying enzymes or proteins necessary for the growth and development of the pathogen. Researchers then screen these proteins against some chemicals or compounds in libraries for potency and inhibitory effect leading to drug candidate identification using computer software after learning the accurate details of the target and lead molecule. This procedure could help pharmaceutical firms, agencies, and research labs avoid following the “false” clues. In contrast to the traditional drug discovery approach, which is time-consuming, expensive, and laborious, a new understanding of the quantitative relationship between structure and biological activity leads to the emergence of computer-aided drug design (CADD) applications in search of new therapeutics against TB. Table 1 shows the advantages of the computer-aided method of designing drugs over the traditional method.

The rapid advances in high-throughput screening (HTS) technologies and computational chemistry created an atmosphere that allows vast libraries of compounds to be screened and synthesized in a short period, speeding up the drug development process [14]. CADD involves storage, management, analysis, and modeling of potential therapeutic compounds. It refers to computational methods and techniques for storing, handling, analyzing, and modeling chemical compounds. It includes computer programs for designing compounds, tools for systematically evaluating possible lead candidates, and the development of digital libraries for researching chemical interactions between molecules, among other topics [15]. Advances in drug discovery involve using computational analysis to identify and validate vulnerable targets, which leads to the emergence of new therapeutics; they are also used in preclinical trials, drastically altering the drug development pipeline. Computational techniques can cut drug production costs by up to 50% [16,17,18]. On average, it takes 10–15 years and $500–800 million to bring a drug to market, with lead analogue synthesis and testing accounting for a significant portion of that cost. As a result, using computational methods during optimization drastically reduces the expenses on drug development, as there are computational models that can screen thousands of compounds before synthesis and in vitro testing.

New therapeutics against TB emerged from HTS techniques and other related software development. There has also been an increase in biological and chemical data available on Mtb to facilitate new target identification. Furthermore, improvements in data storage capacity, supercomputing ability, and parallel processing encouraged the adoption of CADD as an integral component of TB pharmaceutical research. CADD made drug discovery all-encompassing, including different fields. Computational tools of CADD made it possible to ascribe more than 5000 macromolecular structures in the Protein Data Bank (PDB) to Mtb [19,20]. This repository provides a fertile ground for discovering new compounds as potent drug molecules to combat TB [1,19].

**Figure 1:**
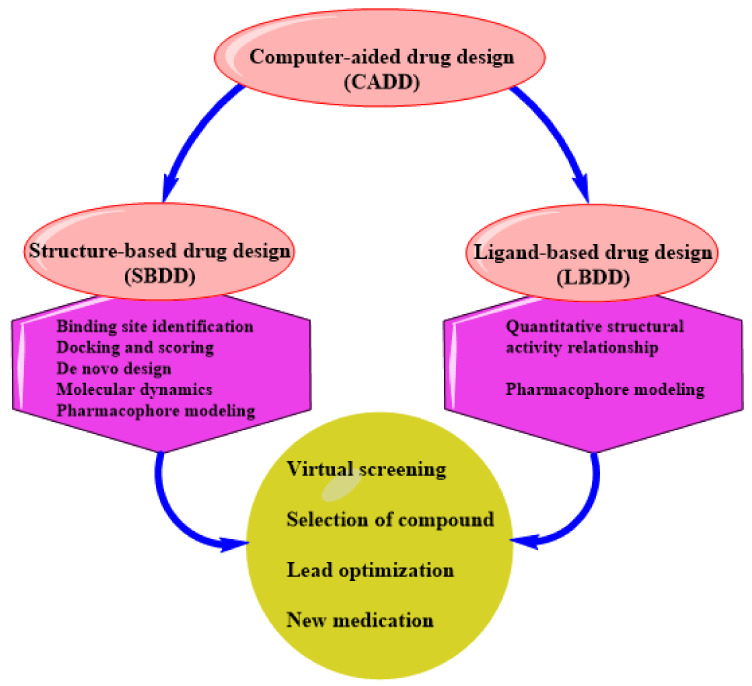
An illustration of CADD.

CADD can be structure-based drug design (SBDD) or ligand-based drug design (LBDD). These are the two most popular approaches to drug discovery (Figure 1). Currently, no single method can meet all the necessities of drug discovery and production. As a result, several computational methods are used widely and effectively in combinatorial and systemic approaches [1]. This review examines the evolution of TB tolerance, current drug management, and the development and adoption of new compounds as anti-tubercular therapeutics. Although there were other recent studies, where developments of TB drugs based on CADD were extensively appraised with respect to specific targets using in silico approaches [21,22], this review provides insight into the most recent developments on the various available resources used in TB drug design and the inclusion and contributions of these resources to the development of new effective therapeutics against Mtb. Similarly, the introduction of machine learning and artificial intelligence to CADD is considered as a new perspective in TB drug management. Our study aids in a better understanding of the current state and the allure of the potentials embedded in using computational drug development for TB.

## 2. TB Pathology, Management, and Control

**Figure 2:**
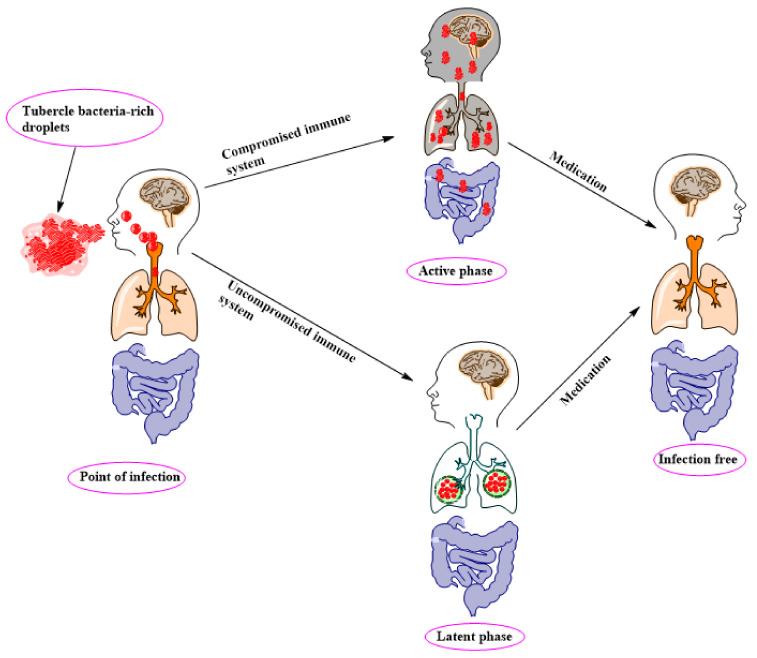
An illustration of tuberculosis (TB) infection phases.

TB is a contagious disease that is transmitted predominantly through the air. An individual becomes infected after inhalation of tubercle bacteria-rich droplets from contaminated air that enters the lungs. The newly infected person may show symptoms due to a compromised immune system from other infectious diseases, such as HIV. However, Mtb remains in a dormant stage if the immune system is not compromised. The alveolar macrophages perceive the bacteria as foreign bodies and internalize them. The bacteria multiply and eventually infect the macrophage, then spread from this point to the entire body system via bloodstream. However, it is crucial to note that infections, such as HIV, and lifestyle diseases, associated with alcoholism and smoking, pose a very high risk in the pathogenesis of TB [23,24]. Generally, TB is considered latent or active (Figure 2). The latent TB infection (LTBI) is not transmissible, and patients in this category show no symptoms. However, patients with active TB can transmit the bacteria, and the common symptoms exhibited by these patients include: fever, weight loss, productive cough, and hemoptysis [25,26]. An estimated 1.7 billion people in the world may contract LTBI and risk developing active TB. A World Health Organization (WHO) report showed that active TB disease affects 5.7 million men, 3.2 million women, and 1.1 million children with 9% of this population also infected with HIV in 2018 [27].

### 2.1. TB Drug Management and Classification

The complete invention and design of the first-line therapy for the treatment of TB in the 1960s was also the first effective treatment and cure of an infectious disease. The classification of anti-tubercular drugs may be according to source, which is either natural or synthetic; mode of action; and situation of the patient (phase or treatment regimen), which are classified as first-line and second-line regimens [28]. Approximately 20 medications are currently available on the market for the management of TB. The use of these therapies can be singly or combined [29]. If left untreated, TB can be fatal. Between 2000 and 2018, physicians rescued approximately 58 million infected people using a traditional regimen. In 2017, there was a report of an 85 percent global success rate in the treatment of newly diagnosed TB cases. However, a 56 percent treatment success rate was reported for drug-resistant TB cases globally in 2017 [27].

### 2.2. First-Line Drugs

**Figure 3:**
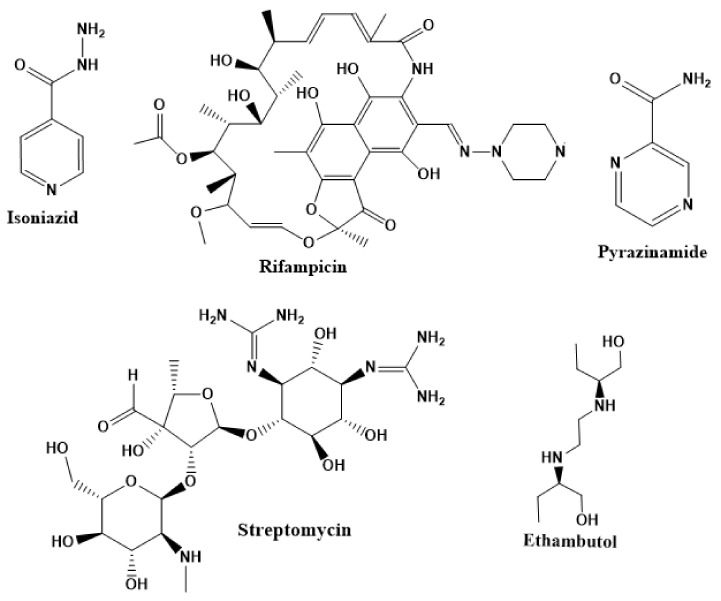
Chemical structures of first-line drugs used in management of TB.

The first-line treatment regimen of TB includes isoniazid, rifampicin, pyrazinamide, streptomycin, and ethambutol (Figure 3) [29]. For latent TB infection (LTBI), the WHO recommends isoniazid alone or combined rifampicin for 3–9 months [30]. Over the years, drug-sensitive TB treatment has entailed the conventional regimen of first-line medications for 2 months, then followed by a combined therapy of isoniazid and rifampicin for another 4 months [2]. Nonetheless, this treatment plan has a high success rate. However, the extended treatment period results in various side effects, such as skin rashes, dizziness, and gastrointestinal disturbance, among others, resulting in patient noncompliance [31].

### 2.3. Second-Line Drugs

**Figure 4:**
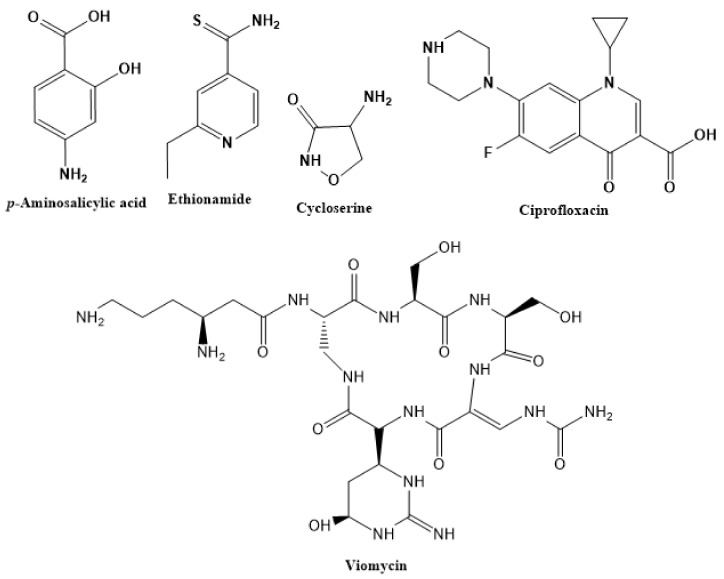
Chemical structures of second-line drugs used in management of TB.

When first-line drugs fail or there is reduced effectiveness, medical practitioners usually introduce second-line drugs. They often prescribe these medications whenever a patient shows signs of drug resistance to one or more medications [32]. The drastic reduction in treatment efficacy that involves incomplete TB treatment regimens often results in disease relapse and resistance. Managing TB resistance entails the fast-tracked development of several drugs to help with global TB control efforts. The second-line treatment regimen includes para-aminosalicylic acid (PAS), ethionamides, cycloserine, viomycin, and ciprofloxacin (Figure 4). These therapies are long-acting, with questionable effectiveness and high toxicity, which also leads to lower compliance and unfavorable outcomes.

### 2.4. Emergence and Treatment of Multi-Drug Resistant TB (MDR-TB) and Extensively Drug-Resistant TB (XDR-TB)

Researchers attributed the emergence of resistance to several factors, including poor compliance to a course of prescribed medications, inconsistent monitoring, medication abuse, and mutations in strains. Resistance can also develop due to change in membrane pumps, changes in the interaction of the drug or target, and chromosome mutations [33]. Another reason may include the low permeability of the mycobacterial cell wall due to its lipid-rich nature, which prevents the accessibility of compounds to targets.

**Figure 5:**
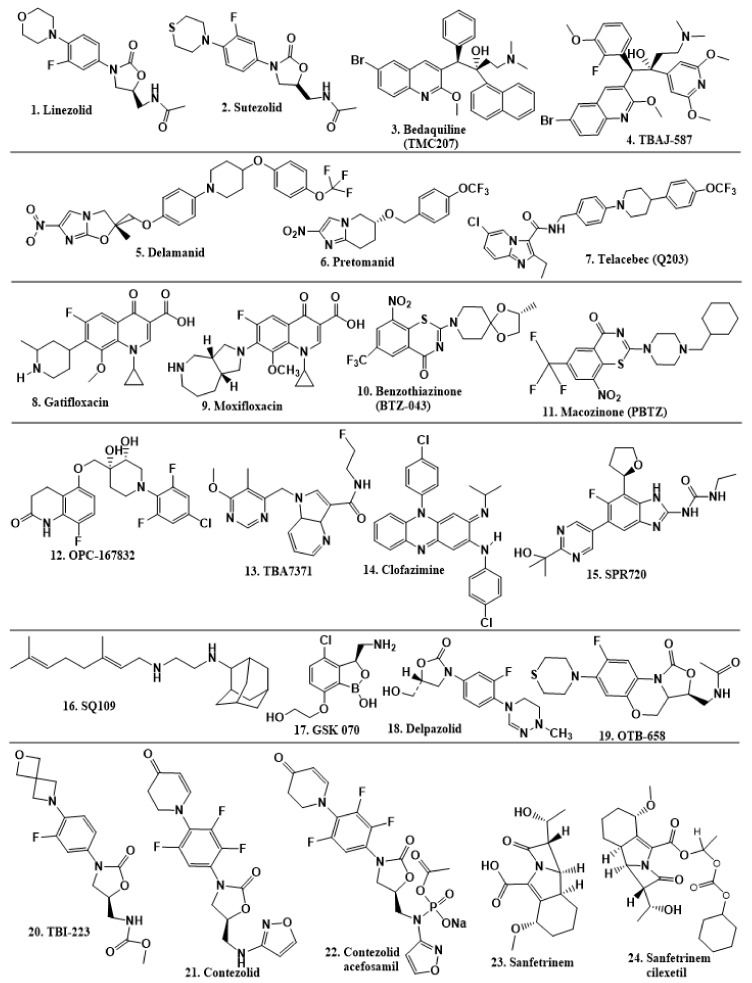
Chemical structures of new Mtb drugs at different clinical trial phases.

MDR-TB was first noticed in the 1990s as the bacteria failed to respond to first-line therapy (isoniazid and rifampicin) [34]. Between 2013–2014, there were reportedly approximately 500,000 new cases of MDR-TB worldwide, resulting in about 200,000 deaths [35]. XDR-TB occurs when both first-line and second-line medications are unsuccessful. Out of the total number of MDR-TB cases registered per year (500,000), 5–7% become XDR-TB [36]. At the end of 2012, the US Food and Drug Administration (FDA) approved bedaquiline (Figure 5 as a medication for resistant TB in response to this emergence [1]. The European Medicines Agency (EMA) granted conditional approval for delamanid (Figure 5) to manage MDR-TB in adults in 2014. The FDA recently approved pretomanid (Figure 5) therapy combined with bedaquiline and linezolid to treat resistant TB [1]. However, this drug has high toxicity and usage associated with an increase in the risk of death. This adverse effect raised concerns about its approval. There was approximately a four-fold increment in mortality among patients managed with bedaquiline during clinical trials compared with those who received the alternative placebo therapy [37]. The threat of drug resistance, on the other hand, is a sobering thought, so there is a need for the development of new therapeutic agents with no cross-resistance to existing treatments.

### 2.5. Current TB Drugs’ Mechanism and Resistance Development

One of the first-line compounds synthesized for TB treatment is isonicotinic acid hydrazide. Otherwise called isoniazid, the compound has a molecular formula C_6_H_7_N_3_O and weight 137.139 g/mol [38]. Although the specific action mode of isoniazid is still a query, researchers proposed several mechanisms. The hydroxyl radical oxidizes INH at the primary nitrogen of the hydrazyl moiety, and the hydrated electron reduction occurs at the pyridine ring, according to a recent analysis by Khan et al., in 2016 [39]. Another related study suggested cell wall penetration of the bacteria via passive diffusion. Subsequent oxidative activation by catalase peroxidase enzyme (KatG) forms a reactive intermediate isonicotinyl acyl radical. This step, preceded by the formation of the INH-NAD, adducts and inhibits InhA (2-trans-enyolacyl carrier protein reductase) of Mtb. Overall, the process limits the formation of mycolic acid, an essential component of the cell wall [38,40,41].

Similarly, other studies proposed the formation of highly reactive oxygen species (ROS) by the KatG-mediated INH activation. The suggested ROS may include superoxide, nitric oxide, peroxide, and hydroxyl radicals and isonicotinic acyl anion. ROS attack different targets in Mtb cells [42,43], causing more oxidative stress and decreasing INH Mtb resistance [44]. INH resistance emerges from a mutation in the KatGat S315T, a catalase peroxidase enzyme [45]. This possibility, confirmed by another study, involved a computational model to understand the mode of action of isoniazid as an anti-tubercular drug. The investigation showed the involvement of the KatG mutation at position 315 (S315T/S315N) in forming hydrogen bonds between INH and mutant Thr(T)/Asn(N) residues, leading to the formation of an INH free radical [46]. Mutations in eight other genes (furA, inhA, kasA, rv0340, iniB, iniA, iniC, and efpA), as well as two regulatory DNA regions (oxyR-ahpC and the promoter of mabA-inhA), were also related to the resistance of INH [45].

Ethambutol (EMB) is a crucial component in anti-TB treatment. It is used in treating Mtb infection in combination with other first-line agents and has a molecular formula of C_10_H_24_N_2_O_2_ and a molecular weight of 204.31 g/mol. The actual mechanism of EMB, like those of some other anti-TB medications, is unclear. Studies showed that EMB inhibits Mtb cell wall synthesis by disrupting arabinogalactan synthesis and inhibiting arabinosyl transferase [47]. EMB resistance is in about 65% of INH-resistant strains [33]. Most cases of EMB resistance are inherent from mutations that occur in the embB gene (mutations in the embC-embA intergenic region (IGR)). Researchers linked resistance to EMB to mutations in the Mtb embB306 gene codon 306, embB406, embA(-16), and embB497 in the majority of cases. There are likelihoods of EMB resistance linked to overexpression and mutations in ubiA [48,49,50].

Rifampicin (RIF) is a natural product with molecular formula C_43_H_58_N_4_O_12_ and a mass of 822.94 g/mol. It is an antibiotic from a Gram-positive bacterium *Amycolatopsis rifamycinica* of the rifamycin group. In addition to Mtb, it has a broad-spectrum antibiotic effective against fungi and viruses. Other derivatives of rifampicin identified over time include rifamycin, rifamixin, rifabutin, and rifapentine. Its activity entails the inhibition of DNA-dependent RNA synthesis. Increasing rifampicin/rifampin use resulted in a mutation in the RNA polymerase b-subunit, causing resistance development. The critical mutant is in the rpoB gene’s codons between 507 and 533, known as the rifampicin resistance-determining region. Codons 516, 526, and 531 are mutated in most rifampicin-resistant cases [51].

Pyrazinamide (PZA) is a compound with the molecular formula C_5_H_5_N_3_O and weighs 123.113 g/mol. PZA is another active medication used in first-line therapy for nearly four decades. It is considered a prodrug with the capacity to penetrate the bacterial cell wall through passive diffusion. It is converted to pyrazinoic acid, its active metabolite, by the action of pyrazinamidase. Pyrazinoic acid inhibits Mtb activity through multiple pathways, including inhibition of the fatty acid synthase (FAS) I enzyme, bonding to the ribosomal protein S1 (RpsA), and membrane inhibition [52,53]. In 1996, the PZA-resistant strain of Mtb reportedly originated in the pncA gene [54]. Similarly, investigators noticed that pncA mutations were responsible for PZA resistance [55]. However, there is evidence on PZA-resistant strains without pncA mutations, suggesting the mediation of PZA resistance by other genes and mechanisms [56]. According to studies, mutated recombinant pncA reduced enzymatic activity, depending on the mutation’s position and form. Any structural defect might potentially impact PZase function significantly [57,58].

Streptomycin (STR), an aminoglycoside antibiotic, has a molecular formula of C_21_H_39_N_7_O_12_ and weighs 581.574 g/mol. It is a natural product effective in the treatment of TB. It comes from *Streptomyces griseus*, a soil actinomycete. STR inhibits protein synthesis by binding irreversibly to the 30S ribosomal subunit and 16S rRNA that codes genes rpsL and rrs [59]. Mutations in gidB, a gene encoding a conserved 7-methylguanosine methyltransferase specific for the 16S rRNA, were identified as the cause of resistance to STR in recent years [60]. The main mechanisms of STR resistance are mutations in rpsL and rrs, which account for around 70% of the resistance observed. A switch in codon 43 from lysine to arginine, which results in high-level streptomycin resistance, is the most widely recorded mutation in rpsL. The most specific mutations in rrs are in between nucleotides 530 and 915 [61]. There are still many streptomycin-resistant strains that do not show mutations rpsL and rrs, which suggests the existence of other possible mechanisms of resistance. 

Ethionamide is an isonicotinic acid derivative that resembles isoniazid in structure. It is also a prodrug that requires activation by ethA-encoded monooxygenase. The mode of action involves generating an adduct with NAD and inhibiting the enoyl-ACP reductase enzyme, which results in mycolic acid synthesis inhibition [62]. Mutations in the etaA/ethA, ethR, and inhA genes lead to ethionamide resistance [63]. Furthermore, experiments with spontaneous isoniazid- and ethionamide-resistant mutants of Mtb indicated that they map to mshA, which encodes a mycothiol biosynthesis enzyme [64].

The first discovery of para-aminosalicylic acid (PAS) was in 1948. It acts by inhibiting thymidylate synthase in iron uptake interference and synthesis of folate. A recent study showed that numerous missense mutations in the folC gene, which encodes dihydrofolate synthase, resulted in resistance to PAS in Mtb isolates [65]. Mutations in the thyA gene associated with PAS resistance were available in clinical isolates resistant to PAS in one research using transposon mutagenesis [66]. Nonetheless, mutations in thyA were found in less than 40% of PAS-resistant strains, suggesting that other drug-resistance pathways may be present [66,67]. 

**Figure 6:**
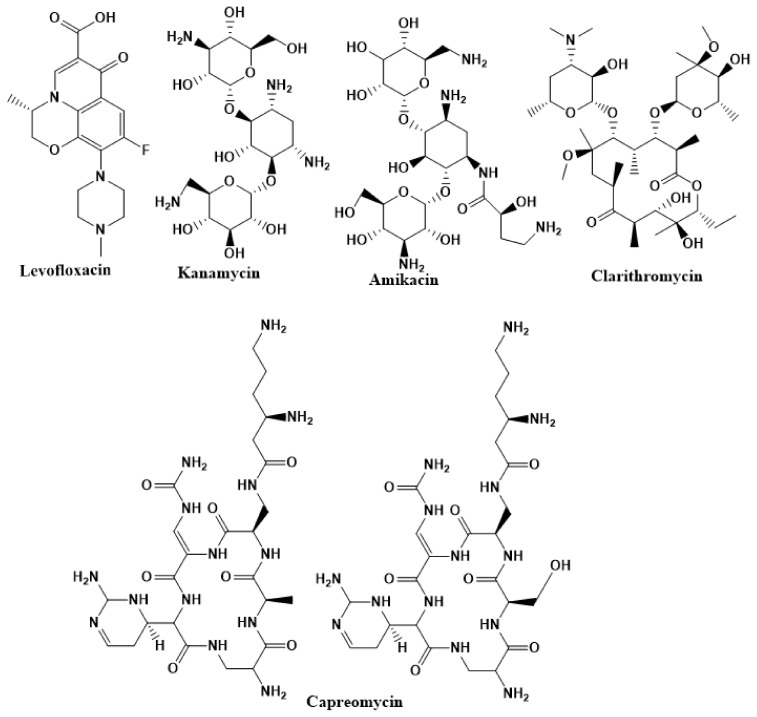
Chemical structures of drugs used in management of resistant TB.

Physicians often prescribe fluoroquinolones (levofloxacin (Figure 6) and melofloxacin (Figure 5)) as second-line drugs for MDR-TB treatment. Fluoroquinolones function by blocking topoisomerase II (DNA gyrase) and topoisomerase IV. These proteins are essential for bacterial multiplication and survival encoded by genes gyrA, gyrB, parC, and parE [68]. Chromosome mutations in the quinolone resistance-determining area of gyrA or gyrB are the most common cause of fluoroquinolone resistance in Mtb. GyrA mutations at positions 90 and 94 are the most common, but mutations at other positions were also identified [69]. Other compounds prescribed (Table 2) for the treatment of TB as second-line drugs and in combination therapy include capreomycin, kanamycin, viomycin, amikacin, cycloserine, macrolides (clarithromycin), clofazimine, and linezolid, among others (Figure 4, Figure 5 and Figure 6).

### 2.6. New TB Drugs Discovered through HTS and Other Approaches

Researchers discovered several TB drugs through the available information when introducing a new compound into the drug regimen. Drug repurposing, drug scaffold modification, revisiting existing targets, target-based screening, and phenotypic screening are among the methods used to discover new anti-tubercular drugs. High-throughput screening applies to Mtb drug discovery, whereby investigators examine compound databases for anti-mycobacterial activity against mycobacterial cells in culture. In most studies, it is rational to establish the potency of the identified hit compounds based on in vitro and in vivo procedures. The development of most recently approved TB drugs and potential agents in clinical trials (Figure 5) entails drug-to-target pathways involving whole-cell screening.

Johnson & Johnson discovered bedaquiline (TMC207 or R207910) by screening around 70,000 compounds against *Mycobacterium smegmatis*. They unveiled the compound in 2004 at the Interscience Conference on Antimicrobial Agents and Chemotherapy (ICAAC), later approved by the FDA around 2012. Bedaquiline inhibits adenosine 5′ triphosphate (ATP) synthase activity and subsequent energy supply, providing unique targeting. Mtb ATP synthase has become a commonly validated target since the discovery of bedaquiline [79].

Pethe et al. [72] uncovered two series of imidazopyridine amides (IPA) from a phenotypic HTS of a library of 121,156 chemical compounds at the Pasteur Institute in Korea for their ability to inhibit Mtb growth in mouse macrophages [80]. The synthesis and evaluation of 477 derivatives of the hit compound resulted in the optimized IPA called telacebec (Q203). The primary target of Q203 is the cytochrome unit bc1 complex, a critical component of the electron transport system necessary for ATP synthesis [81]. Qurient Co. Ltd. is currently conducting a phase 2 clinical trial to assess the bactericidal efficacy, safety, tolerability, and pharmacokinetic properties of Q203 in repeated oral doses. Other compounds discovered through HTS include benzothiazinone and maconizone, azaindoles, and OPC-167832.

Further chemical research on some existing antimicrobial molecules through modification of drug scaffolds led to the development of many analogues, including pretomanid and delamanid. These compounds were recently recorded as anti-TB drugs and are constituents of the new multi-drug resistant (MDR) regimen. Delamanid (OPC-67683) and pretomanid (PA-824), both nitroimidazoles, were discovered in *Streptomyces eurocidicus* [82]. In 2014, the European Medicines Agency (EMA) granted conditional approval of delamanid, a nitro-dihydro-imidazooxazole derivative. Otsuka Pharmaceutical developed delamanid for managing MDR-TB in adults. Delamanid and pretomanid have a similar multi-target mode of action, influencing biosynthesis of the cell wall via disruption of methoxy- and ketomycolic acid synthesis, as well as respiratory toxicity through nitric oxide release during bacterial drug metabolism [83].

Pretomanid is another analogue that shows activity against Mtb [83]. PathoGenesis Corporation, under the aegis of the Global Alliance for TB Drug Development, discovered pretomanid. In animal models, this relatively small molecule demonstrates excellent in vitro and in vivo activity and seems healthy and well-tolerated. The mode of action is through nitroreductase activation, which hinders the synthesis of proteins and cell wall lipids [84]. More clinical trials are currently being conducted on the drug. The FDA approved a review of a new drug application for pretomanid recommended to treat XDR- and MDR-TB in conjunction with bedaquiline and linezolid.

The search for a new medication as a second-generation drug from ethambutol led to creating a library containing 63,238 compounds based on 1, 2-ethylenediamine pharmacophore. These compounds, screened against Mtb, resulted in the potent SQ109 discovery through a joint effort by scientists from Sequella, Inc. (Rockville, MD, USA) and the US National Institutes of Health [85]. The mode of action of SQ109 involves inhibition of MmpL3, which is a membrane carrier for trehalose monomycolate involved in cell wall synthesis. It also inhibits MenA and MenG, which are essential enzymes in the biosynthesis of menaquinone. SQ109 acts as an uncoupler by reducing ATP synthesis [86]. 

Contezolid and contezolid acefosamil (Figure 5) are designed by modification of the linezolid scaffold to overcome the limitations associated with its clinical use, which include myelosuppression and serotonergic monoamine oxidase inhibition. Contezolid is currently in phase 3 clinical study and its intravenous administration is facilitated by the introduction of its water-soluble prodrug called contezolid acefosamil [70,87], which has no appreciable antimicrobial activity. The in vitro activity of contezolid against resistance Mtb is related to that of linezolid [71,88].

Sanfetrinem and its oral prodrug sanfetrinem (Figure 5), cilexetil, are novel carbanepem introduced by GSK in clinical study. The discovery of the drug was through screening nearly 2000 β-lactams against Mtb H37Rv. It was also investigated against MDR and XDR clinical isolates promising activity. Other new TB drugs in different clinical trial phases are available at www.newtbdrugs.org (accessed on 7 November 2021) [72]. Table 2 shows some new Mtb drugs at different clinical trial phases.

### 2.7. Protein Target in Mtb Drug Design

**Figure 7:**
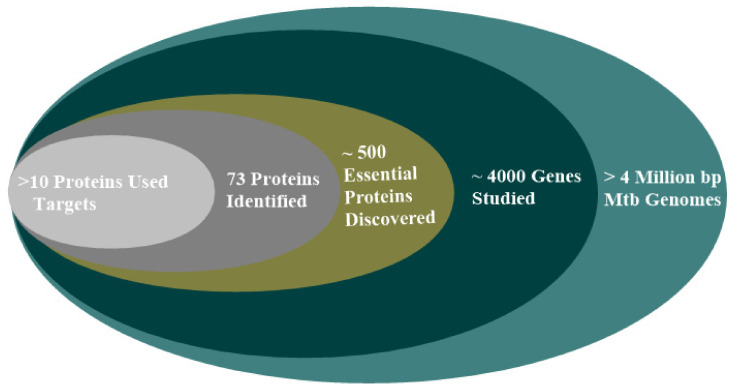
A representation of the genome of Mtb genes, essential proteins, and the number of proteins currently in use as targets for drug discovery, redrawn from the literature [91].

In recent times, the development of new Mtb therapeutics entails identifying compounds that effectively inhibit specific targets essential for the bacterium survival and proliferation in the host. Mtb is known to secret essential proteins that: aid the acquisition of its nutrients, alter the host immune system, and help to develop resistance against therapeutics [89,90]. These constitute the crucial aspect of host–pathogen interactions. So, inhibiting any of these essential proteins disrupts pathogen activities in the host and limits their devastating effects on the host. Inhibition has been the dimension adopted in drug design. Computational methodologies enabled the screening of several libraries of compounds against some essential proteins known for Mtb survival. Despite more than 500 discovered essential proteins of Mtb, there are only 73 established targets and 10 potential targets in the discovery of new anti-tubercular drugs (Figure 7). Therefore, there is a need to explore the inhibition of several other essential proteins for identifying new effective medications against Mtb [91]. Some scientists opine that a suitable drug should have the capacity to inhibit multiple protein targets that restrict the possibility of the pathogen building resistance over a long period. Such an approach reduces the complicated regime associated with the treatment of Mtb infections.

## 3. SBDD as an Indispensable Tool in Computational Drug Design

Like target discovery and identification, novel drug development through identifying compounds that can inhibit protein targets is critical in the drug discovery process. The traditional method of identifying potential leads is through experimental HTS, which is laborious, time-consuming, and relatively expensive [92]. A typical drug research cycle might take up to 14 years [15] and cost about USD 1 billion [93] from the point of target identification to FDA approval. With this traditional method, there has been a recent decline in the number of new medications entering the market due to failures in various phases of clinical trials [94], despite the invested time and limited available resources. In November 2018, a study [95] showed the estimated total cost of pivotal trials for developing novel FDA-approved medications. According to the American Pharmaceutical Association, the median amount for effectiveness studies of the 59 new medicines authorized by the FDA in 2015–2016 was USD 19 million [95]. As a result, it is critical to overcome the constraints of current drug discovery approaches with computational alternatives that are efficient, low-cost, and broad-spectrum in nature.

Conversely, rational drug design is more efficient and cost-effective than the traditional drug discovery technique (classical or forward pharmacology). Reverse pharmacology is another term used to describe the drug design and discovery process. The initial step is to identify and confirm the target proteins, then use them to screen small-molecule data libraries [96]. This concept benefitted from significant progress made in structural and molecular biology and improvements in biomolecular structural identification techniques. More than 100,000 proteins had their three-dimensional (3D) structures determined using these approaches [97]. In combination with the storage and appropriate organization of large amounts of data, there is a lot of excitement about developing sophisticated and robust computational methods.

**Figure 8:**
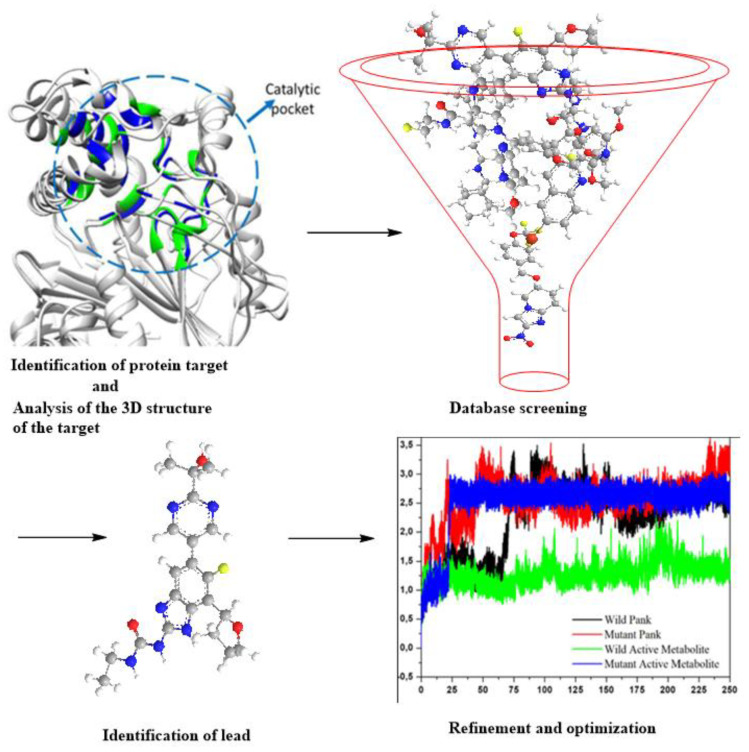
An illustration of the SBDD process.

The completion of the Human Genome Project and advancements in bioinformatics led to the availability of target proteins. These target proteins enhanced the pace of drug development, laying the background for developing SBDD. SBDD is an increasingly important component of industrial drug development initiatives and academic research [98,99]. It is a more precise, time-efficient, and hands-on approach for lead identification and optimization (Figure 8). The most often utilized computational approaches in SBDD include structure-based virtual screening (SBVS), molecular docking, and molecular dynamics (MD) simulations, which are all examples of applicable techniques. These approaches have several applications, including binding energetic predictions, ligand–protein or protein–protein interactions study, and evaluating conformational changes [100]. The current advances in the bioinformatics and cheminformatics business were obtainable through a significant increase in the number of software packages designed to facilitate the efficient discovery of new drugs. However, it is critical to select exceptional packages to ensure a successful SBDD process [101]. The automation of all phases in an SBDD process reduced the SBDD timeframe [98]. The availability of supercomputers, computer clusters, and cloud computing also aided in speeding up the discovery and assessment of potential leads.

### SBDD in Drug Discovery and Design

SBDD is the most potent and effective procedure in the entire drug discovery and development framework. In the drug discovery process, computational resources are a valuable tool for speeding up the process. The steps include various screening procedures, combinatorial chemistry, and computations of drug properties, such as absorption and distribution, metabolism, excretion, and toxicity (ADMET) [102]. SBDD is a monotony sequence process that continues through several cycles to develop a therapeutic candidate optimized for clinical trials. There are four stages in the drug discovery process, namely, research and development, clinical trials, registration, and the regulatory submission phase.

The first phase involves the identification of a possible therapeutic target, as well as efficacious ligands. Cloning the target gene and subsequent extraction, purification, and three-dimensional structure identification of the protein are essential steps in this procedure. The next stage is to identify the 3D structure of the target receptor in combination with the potential ligand discovered in the first stage. Structural perspectives into the ligand–protein interaction aid in analyzing various binding geometries, identifying binding pockets and ligand–protein interactions, and interpreting conformational changes resulting from ligand–receptor interaction and detailed mechanistic and dynamics studies [97]. As a result, there is an improvement in the effectiveness and specificity of the lead via many iterations. The third phase entails clinical studies/trials of the lead compounds and is marked complete if successful. The fourth step is when the medication is approved for release into the market and made available for clinical usage.

A variety of computational techniques adopted to dock small molecules or fragments of compounds from large databases enables inserting molecules into the catalytic site of an enzyme. The scoring function allows determination of the relative importance of these molecules in terms of their steric and electrostatic interactions with the catalytic site of the protein. With the 3D structure of the target molecule, it is possible to conduct in-depth research into the electrostatic characteristics of the active domain and determine whether there are cavities, apertures, or allosteric pockets. Recent SBDD techniques consider the essential features of the binding pocket of the targeted receptor to develop effective ligands [103,104]. The top hits are synthesized and optimized in the second step of the process. The biochemical tests used to evaluate the top-ranked compounds with high affinity for selective regulation of the target enzyme are also performed in vitro on the compounds with the highest affinity [105]. Because these ligands interact with critical cellular processes, the creation of pharmaceuticals with the desired therapeutic and pharmacological impact is facilitated [106]. Experimental techniques used to examine the biological characteristics of the chosen compounds include their effectiveness, affinity, and potency [107].

SBDD is a computer approach that pharmaceutical firms and researchers frequently utilize. The SBDD approach led to the discovery of many medicines that are now available on the market. The incredible success story of SBDD so far was the approval of HIV-1 inhibiting drugs by the Food and Drug Administration (FDA) [108]. SBDD medicines include raltitrexed (a thymidylate synthase inhibitor) [98] and amprenavir (an HIV protease) [108,109]. Norfloxacin (an antibiotic) [110] and isoniazid (an anti-tuberculosis) stem from pharmacophore modeling and virtual screening (VS). Other instances of successful drug discovery using SBDD methods are available in Table 3. Despite the success story of SBDD, there exist limitations in the available SBDD techniques because of the failure scenarios that they encounter. Even though the SBDD workflow contains various efficient approaches, they all have limits that necessitate further study.

## 4. Status of Computational-Aided Drug Design and Discovery in TB

The drug discovery process for novel anti-tubercular therapies has evolved throughout the years due to the accumulation of biological and chemical data, the identification of numerous validated targets, and the advancement of high-throughput screening methods and software algorithm development. Aside from that, advances in data storage capacity, supercomputing power, and parallel processing allowed computer-aided drug design (CADD) to become an integrated component of TB drug design and discovery research during the last several years. As computing power continues to grow, it may soon be possible to conduct extensive exploration of the vast chemical space, which is estimated to contain about 1060 organic molecules below 500 Da, to identify potential therapeutic attractive moieties [124] for effective Tb treatment. 

**Figure 9:**
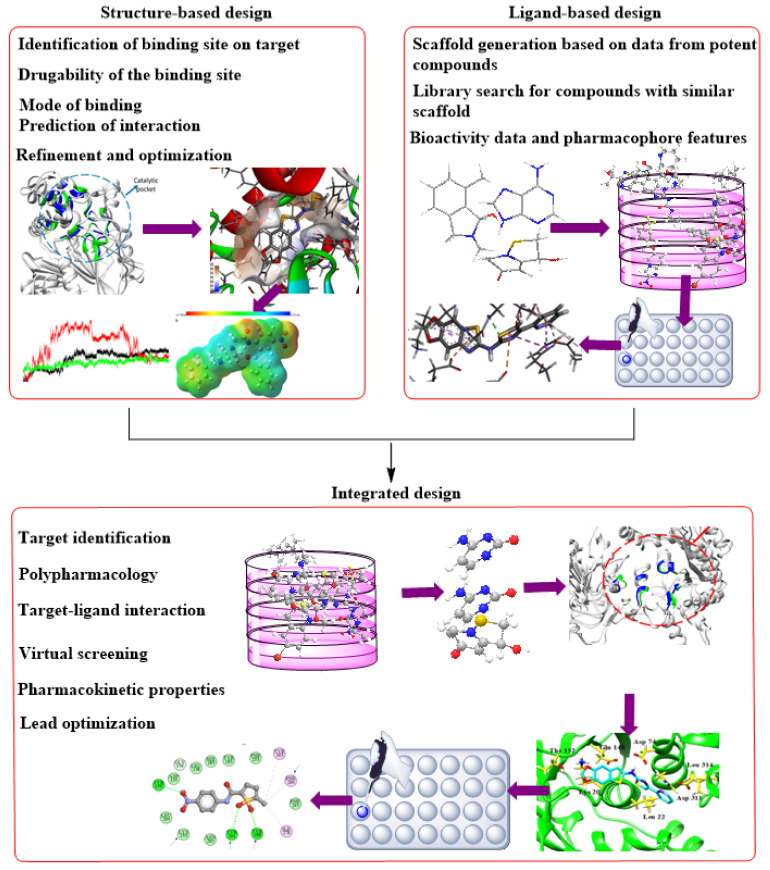
Complementary integration of Structure–Based Drug Design (SBDD) and Ligand–Based drug Design (LBDD) approaches.

Furthermore, the massive protein structural data, which includes more than 180,000 macromolecular structures available in the PDB (www.rcsb.org accessed on 8 November 2021) and other protein repositories, gave the computational SBDD (Figure 9) concept an impetus. The pulled structures allow identification of critical receptor catalytic and allosteric sites, molecular nature, and crucial features for in silico SBDD research. There has been much focus on TB with the countless ongoing drug discovery research, with several thousand published CADD studies. Although this is the case, Ekins et al. [125] identified gaps in the application of computational methods in TB research, resulting in a slow stream of candidates’ drugs entering the TB drug pipelines, despite the evident need and immediacy for an effective treatment against this infection. Therefore, there is a need for more rigorous efforts to develop TB drugs leveraging the benefits provided by computational techniques.

Methods based on computation or in silico are currently burgeoning and knowledge-driven, systematically evaluating existing data to explore protein function and develop novel compounds that can modulate its activity. Depending on the availability of protein structures, computational drug discovery techniques are typically SBDD and ligand-based drug design (LBDD). To enhance the success rate of current drug development initiatives, it has been standard practice in the pharmaceutical industry to integrate these approaches in a complementary manner with one another (Figure 9). When using SBDD, it is necessary to have a three-dimensional (3D) model of the target protein to evaluate and exploit the druggable pockets for screening and creating appropriate ligands, which can subsequently be experimentally confirmed and enhanced. Instead of relying on protein structural data, LBDD uses the information obtained from a wide array of ligands with proven activity to develop prediction models for hit and lead optimization [126].

Different SB and LB tactics, or a mix of them, might be used at different phases of TB drug design, discovery, and development to mitigate the difficulties associated with experimental techniques. With the availability of the TB genome and proteome and a wealth of structural information, researchers can use big data and molecular simulation to identify potential targets for treatment vs. allows choosing the most promising prospective candidates from a database comprising millions of compounds for a specific TB target. From the validated candidates, a quantitative structural activity relation (QSAR) study is obtainable to understand the mechanism of action and ADMET properties. QSAR facilitates compound development with improved efficacy, as well as pharmacokinetic and pharmacodynamics properties.

The information gathered from this research (both positive and negative outcomes) may be saved and used for additional iteration and technique optimization in designing novel TB drugs in the future. Structure-based vs. produced many anti-tuberculosis compounds with appreciable enzymatic inhibition (Table 4 and Table 5). This study provides an overview of the SBDD process and the current approaches for TB drug development in the modern era. Furthermore, we provide an insight on the machine learning (ML) techniques designed to accelerate the process, procedures, management, and application of large amounts of data in TB drug design.

## 5. Data Application and Management in Tuberculosis Drug Development

Massive data and complex data analysis are the hallmark of the fourth industrial revolution (4IR), profoundly impacting our daily lives’ coordination and conduct. The rise of a big data approach transformed our strategies to deal with age-old challenges in tuberculosis drug development through innovation in cloud data storage and management and improvement in bioinformatics and cheminformatics algorithms. Furthermore, the affordable sequencing technology enables studying all aspects of molecular characters of diseases. Examples are epigenetics, RNA sequencing, metagenomics, targeted sequencing, whole-genome sequencing, and variant detection sequencing [146]. SBDD and other forms of drug development leverage the analysis of vast biological and chemical data generated and stored on publicly available database repositories in cyberspace [147]. 

Considering that tuberculosis is a long-standing disease, volumes of accumulated information await usage to curb this infection. Information on TB drug development is available on the TB Database (http://tbdb.bu.edu/tbdb_sysbio/MultiHome.html; accessed on 5 September 2021) [148,149]. Similarly, Mycobrowser (https://mycobrowser.epfl.ch/; accessed on 5 September 2021) [150] contains information on mycobacterium multi-omics. This repository [150] stores experimental and computational models of TB molecular mechanism pathways and several pathogenic mycobacteria. Mycobrowser also connects with UniProt (https://www.uniprot.org/; accessed on 5 September 2021) [151], the most widely used protein database containing mycobacterium protein information. Clinical data on TB are also accessible on the TB Portals (https://tbportals.niaid.nih.gov/; accessed on 5 September 2021) [152]. Innovations in structural biology and bioinformatics resulted in an influx of structural data. These interventions led to thousands of 3D protein structures generated from X-ray crystallography, nuclear magnetic resonance (NMR), cryo-electron microscopy (cryo-EM), and homology modeling experiments. PDB [19], PDBsum [153], and other structural databases store these research results. Hence, the availability of chemical libraries (Table 6) was made possible by expanding the digital chemical space [124] and advancements in chemical synthesis [154].

### 5.1. SBDD Based on Mtb Proteins

The availability of therapeutically important protein 3D structures made SBDD the most desirable approach for drug design and development ahead of ligand-based drug design (LBDD). However, to enhance the success rate of recent drug development initiatives, it has become customary to integrate SBDD with LBDD approaches in a complementary manner. Using the 3D structures of targets to study and exploit the catalytic pocket, SBDD can search and create appropriate ligands that can subsequently be verified and optimized experimentally. To mitigate the difficulties associated with experimental techniques, several types of SB and LB tactics, or a mix of them, might be used at various phases of TB drug design and development. With the availability of TB multi-omics and a large amount of structural biodata, we can use cheminformatics data mining, data engineering, docking, and homology modeling to identify potential targets.

Virtual screening facilitates choosing the most promising prospective ligand(s) from a database comprising millions of identified molecules for a specific tuberculosis target. The output of candidate compound validation using structure-activity (SA) studies enables a better understanding of the mechanism of action and ADMET (absorption, distribution, metabolism, excretion, and toxicity) properties, thus allowing better development of compounds with improved activity and better pharmacological profiles. The information gathered from this research (both good and negative outcomes) may be saved and used for additional iteration and technique optimization in the future design of new tuberculosis drugs. More than 800 CADD software and webservers (free or commercial) are available, hosted by the Swiss Institute of Bioinformatics at www.click2drug.org (accessed on 5 September 2021). These provide unlimited opportunities to explore drug discovery and design [1]. Table 7 summarizes some available CADD software.

SBDD takes advantage of target protein 3D structure availability. However, if the 3D model of the therapeutically important receptor is not available, computational approaches through homology modeling enable the 3D model prediction of the receptor. Homology modeling or comparative modeling is the most reliable method for 3D protein structure prediction. The methodology entails predicting the 3D structure of the receptor from a homologous protein with at least a 40% similarity index. Threading and ab initio modeling are also methods of protein structure prediction [15]. After obtaining the 3D structure of the target, it is crucial to validate the model by examining the molecular characteristics in a Ramachandran plot. This metric shows the distribution of the ϕ and ψ dihedral angle conformations of the constituting residues in the receptor structure [179]. There are several techniques to validate the predicted protein model [15,175,180].

After determining the target structure, the next step is to determine the catalytic pocket. Catalytic or binding pockets are tiny spaces where ligands attach to the target, inducing the intended result. Consequently, it is crucial to identify the most suitable location on the target protein for ligand binding. Even though protein is dynamic in nature, only a few techniques can identify possible binding residues in the binding pocket. Identification of binding sites on a particular target requires the knowledge of interaction energy and van der Waals (vdW) forces. There are many strategies for catalytic site mappings using interaction energy computation through SBDD. This technique can identify locations on the target receptor that interact positively with functional moieties on the drug-like compounds. These approaches find probes that have energetically advantageous interactions with proteins. Q-SiteFinder [176] is an energy-based technique for predicting catalytic sites widely utilized in the pharmaceutical industry. It is possible to compute the vdW interaction energies of proteins with a methyl probe by using this approach. Those with favored energy values are maintained and grouped in the final product. The total interaction energies of these probe clusters serve as the determinant of their ranking. Aside from that, the functional annotation of interacting protein residues in the binding site allows for the determination of the binding site.

It is also essential to remember that additional possible binding sites, referred to as allosteric sites, may also be present on the target protein surface. Drug development attempts in the conventional sense frequently target the important (orthosteric) binding site to prevent natural substrate binding. Besides, researchers have unveiled noncatalytic sites of Mtb proteins. Shi and colleagues identified a second druggable binding site (allosteric) in Mtb UDP-galactopyranose mutase (UGM) [181]. MS-208, a well-known Mtb-UGM inhibitor, was categorized as a noncompetitive/mixed inhibitor based on NMR and kinetics investigations. This observation implies that MS-208 binds to another location in the receptor and affects the natural enzyme substrate from recognizing the primary pocket. They [181] predicted the allosteric sites for MS-208 on the enzyme via docking with AutoDock Vina [182]. The two identified regions, designated A-site and S-site, show favorable and stable interaction with the ligand after molecular dynamics using Amber [183]. Simulations facilitate structural and functional relationship determination. Because the A-site-bound structure demonstrated the most stable complex formation with good interaction energy and a higher number of contacts, they hypothesized that this site represents an allosteric druggable binding site in Mtb-UGM [181].

After appropriate identification of all druggable sites on the receptor, next comes hit discovery, accomplished by docking chemical libraries into the active cavity of the target receptor. Earlier, the routine in lead discovery required choosing a specific collection of ligands that can play a critical role in identifying and optimizing leads [177]. SBDD blends two distinct approaches for hit search (VS and de novo design) into a single framework.

### 5.2. Virtual Screening as a Method of Lead Identification

Currently, vs. has emerged as a dynamic and profitable technique in the pharmaceutical business, particularly for prospecting new drug-like compounds or so-called lead identification [184]. There are two forms of VS: ligand-based vs. (LBVS) and structure-based vs. (SBVS). Biological data is processed in LBVS to distinguish inactive molecules from active ones. Based on consensus pharmacophores, this information facilitates highly functional scaffold identification [185], similarity, or various descriptors. LBVS produces results that are closely related to known active pharmaceutical ingredients. The procedure involves scanning chemical libraries of structures to find molecules with known like potency or that share a pharmacophore or moiety with known activity. The results are typically positive (pharmacophore substructure similarity search) [186]. A moiety substructure search requires using the 2D- or 3D-structure of various ligands to find closely related structures. Usually, comparable substances have similar effects when using ligand-based techniques; thus, they are called similarity methods. For example, if one or more active compounds are known, it is feasible to search a database for comparable but more potent compounds [187].

SBVS allows docking numerous chemical compounds against an enzyme-binding or catalytic site in a short time [188,189,190,191]. The computer algorithms facilitate target protein docking with one of the vast libraries of drug-like chemicals that are commercially or publicly accessible (Table 6). Subsequent steps for search refinement are molecular docking, MD simulations, and experimental tests to obtain IC_50_ or other efficacy parameters [192]. SBVS relies on the scoring of ligands to function correctly. In contrast to ligand-based techniques, structure-based techniques do not rely on previously collected experimental data to be effective.

### 5.3. De Novo Drug Design—A Signature to the Drug Discovery Process

De novo drug design involves creating unique chemical compounds from the ground up, starting with molecular building blocks. The essence of this technique is to design chemical structures of tiny molecules that bind to the target active site with high affinity [193], then test these structures experimentally. A variation in approach is typically employed when designing from scratch, and the design algorithm must integrate the search space information acquired. Usually, researchers incorporate positive and negative designs with one another. When using the former strategy, a search is to constrain certain regions of chemical space, which increases the likelihood of discovering results with notable characteristics. The search parameters are set in the negative mode to avoid choosing false positives [194]. Despite its sufficiency in functional scoring analysis, chemical compound design using computational approaches connects organic synthesis but cannot replace it [195]. It is fundamental in the design stage to conduct a thorough evaluation of candidates’ compounds. One of these evaluation tools is the scoring function; multiple scoring functions for multi-objective drug discovery hybrids [196] create many different characteristics simultaneously.

De novo drug design is in two categories: (A) ligand-based drug design and (B) receptor/enzyme-based drug design. The latter method is popular currently. Creating appropriate small molecules for enzyme-based design requires high-quality target protein structures and precise knowledge of proteins’ active sites. The approach entails small molecules designed by matching fragment moiety into the target proteins’ binding pockets. The process requires using computer programs or co-crystallization of the ligand with the receptor [197]. Two ways to execute the ligand-based design are by linking together crucial components, such as atoms or fragments (single rings, amines, and hydrocarbons) to produce an entirely new chemical molecule, or by simply generating ligands from a single parent unit. The fragment-linking technique uses information of the active site to map the likely interaction locations for the different functional groups contained in the design drug fragments [198,199]. One must link these functional groups’ moieties to one another to form an absolute compound. The fragment-growing method features fragment development within the active site, monitored by appropriate search algorithms [199].

These search algorithms make use of scoring systems to determine the likelihood of growth. Fragment-based de novo design is a method of creating new molecules that use the whole chemical space. When using the linking technique, the selection of linkers is imperative. The outside-in strategy and the inside-out approach are both methods for anchoring fragments in the binding site. The outside-in system is the more common method. The outside-in methodology involves the construction blocks placed near or on the edge of the binding site, and the active site gradually expands inward. The inside-out method uses construction pieces randomly placed within the active site region, then constructed outward [100].

### 5.4. Molecular Docking and Density Functional Theory Applied to Mtb

Molecular docking has been a prime computational technique of SBVS against Mtb enzymes. The molecular-docking technique was the subject of many published research articles as a tool in drug design against Mtb (Table 8). According to the Himar1 transposon mutagenesis study conducted by DeJesus in 2017 [200], the majority of the enzymes targeted by this method are enzymes encoded by crucial genes, with the exclusion of antigens BioA, NarL, 85c, EthR, and LipU. Although this technique assigns nonessentiality to genes based on in vitro growth, it cannot be relied on to determine whether genes are nonessential in vivo [201,202]. For instance, the NarL enzyme is necessary for anaerobic survival throughout infection, while BioA is crucial for biotin synthesis during the latency phase of Mycobacterium TB infection [203,204]. Furthermore, the EthR protein functions in developing ethionamide resistance and, consequently, survives potentials after drug treatment [205,206].

Many studies of the different targeted enzymes indicate that many are engaged in either intermediate metabolism or lipid metabolism in Mtb. In addition, DNA and RNA regulatory enzymes and cell wall regulator proteins make up the remaining target proteins. Researchers show at least three SBVS efforts against DprE1 and InhA, with InhA being the most frequently targeted. Studies show that InhA is the ultimate target of both isoniazid and ethionamide once activated [218]. As a result, InhA provides a validated target whose suppression has an in vivo influence on the survival of Mtb. Also, numerous antimycobacterial medicines target DprE1 in the current anti-TB research pipeline [29,59]. PyrG, a newly confirmed TB target, also attracted the attention of researchers [219]. The chemicals used in most research works (Table 8) are from generic chemical databases containing millions of compounds, while TB-specific databases and natural product, therapeutic repurposing-focused, and other libraries comprise other chemical compounds reported. As a result, the focus of these early drug discovery initiatives continues to be on totally new drug-like chemical discoveries. The apparent lack of further experimental evidence (in vitro or in vivo) showing compound bioactivity in many of these investigations (Table 8) is an evident issue that precludes these anticipated compounds from being carried onward [206].

Another important computational tool in drug discovery is the density functional theory (DFT), which applies to TB research for the investigations of catalytic processes [220,221], structure-activity relationship analysis [222], and inhibitor potency [192,223]. Chi and colleagues [223] adopted DFT in an anti-tubercular study to confirm their first observations of a change in an inhibitor-binding mechanism in the MbtI protein after adding a substituted enolpyruvyl moiety to the parent chemical structure previously generated from isochorismate. From their [223] observation, there were two distinct binding mechanisms (states 1 and 2) noted in the X-ray crystal structures of MbtI complexed with its inhibitors, indicating that the active site is flexible enough to permit ligand binding. With the aid of Gaussian 09 software application [224] and a theoretical-level hybrid B3LYP [225,226], they computed the global minimum configuration of the (E)-3-(1-carboxyprop-1-enyloxy)-2-hydroxybenzoic acid (AMT), Z-methyl-AMT, and E-methyl-AMT inhibitors complexed in solution. The results revealed that the global minimum geometry of both free Z- and E-methyl-AMT is comparable to its bound geometry (state 2), showing that its arrangement enables binding to MbtI. The computation of conformational entropy quantities for the three molecules indicated that Z-methyl-AMT is the least disorganized. Z-methyl-AMT has a conformational lock provided by the methyl moiety in its structure. Even though a pure Z-isomer has not yet surfaced to distinguish it from the E-isomer empirically, this discovery [224] justifies the powerful interaction of methyl-AMT to MbtI. It provides further knowledge for the future creation of new and effective MbtI drug-like compounds with the aid of DFT.

Despite the widespread success and popularity of DFT, it has flaws stemming from the approximations employed in its operational mode. DFT is difficult to use for system descriptions mainly composed of dispersion (van der Waals) forces, such as gaseous systems, or systems in which dispersion contributes significantly, such as biomolecular systems [227]. Thus, numerous research studies examined the incorporation of van der Waals [228,229,230] to improve performance and enhance this technique. In addition to these constraints, the description of global potential energy surfaces of charge exchange excitations [230] is a prime restriction of DFT use in computational drug design. DFT usage overly favors sophisticated users and requires thorough reviews to determine the level of theory/methods to use for a particular system.

### 5.5. Advantages and Drawbacks of Computational Methods

The PDB [19] contains more than 2500 enzymes specifically on TB [206], and a good guess is that there are likely thousands of ligand possibilities. The protein and ligand wealth of data are immediately accessible on the Internet with a click. To better understand TB proteins and their functionality, it is now feasible to examine them to know how to inhibit them at the molecular level [231,232,233]. Even with computational technique advancement for drug development, it is critical to remember that they are different from experimental approaches; every methodology has certain limits that rely on the system and all other relevant aspects of the studies [234,235,236].

The flexibility and efficiency of ligand-based drug design are the two most significant advantages of this approach. LBDD indeed has a lengthy history and many identified candidates, despite the absence of structural knowledge about the protein [237,238,239]. Despite this, the uses of ligand-based techniques require considering several factors. First and foremost, ligand poses with the lowest conformational energy, which is frequently different from the similar bioactive compound conformation [240,241]. LBDD requires that ligands interact at the same active site and exhibit a similar conformation [240,241]. Second, for drugs to be deemed alike, they must be assessed using the same method and conditions [242]. In a third instance, the occurrence of activity cliffs [243,244,245] induces questioning the fundamental premise that “similar structures show comparable activities.” Hence, while picking possible drug-like candidates from a pool of hits, investigators must exercise caution during selection. Finally, considering the investigation aims, there are challenges in measuring the impacts of solvation and enzyme flexibility. The advantages of using SBVS for potential inhibitor discovery for a specific TB target [231,233] include reduction in cost, time, and effort dissipated. The most promising hits identified go through further experimental investigation and drug development. SBVS finds its best application in screening millions of molecules for drug-like molecule identification.

On the other hand, the optimization and validation of the iteration approaches of computation in virtual screening drug design and development are far from flawless. The process is reliant on the enzyme system and chemical moiety utilized, resulting in prejudice in the in silico model. The constraints associated with different models make it difficult to establish that one approach is superior to the other. Many research findings have been published in this area [246,247]. Another limitation of using computational study in drug design is the computational cost inherent in incorporating protein mobility and solvent parameters [126]. For instance, vs. methods disregard tautomerism and the protonation effect. However, it considers the ionization level of the molecules, thus potentially missing some lead hits. Fortunately, emerging computational systems are keeping up with these drawbacks. Improved sampling methodologies, high-performance computing, molecular dynamics, and multidisciplinary drug development frameworks are examples of new drug discovery initiatives. These approaches enable simulations for a variety of enzyme targets in nanoseconds [248,249,250]. Despite several advancements and current advances in SBDD, a reliable solution is yet to be uncovered and implemented in practice. For some difficulties related to water molecule (solvation) inclusion and the flexibility of an enzyme molecule to be resolved, they require breakthrough algorithms for accuracy [191].

## 6. Conclusions and Future Perspective

Tuberculosis continues to be a significant public health issue worldwide, prompting the rapid discovery and development of new antimycobacterial medicines. CADD has facilitated TB drug design and discovery. Currently, computers in medicine are a renowned cornerstone in drug discovery and development research. Theoretical and computational methods remain vital tools to search for potent therapeutic leads despite the harsh and unfair criticism around their usage. There are successes achieved through various software innovations in drug discovery and development pipelines, particularly in antitubercular drug discovery and development. Continuous improvements in the capacity of computer and software availability can improve the effectiveness of current computational tools and their applications in various phases of the drug development pipeline. While these approaches are plausible, they are not bulletproof because each instrument has limitations and approximations frequently employed during the analytical process. Therefore, it is advisable to combine several in silico techniques to avoid bias from using one software. The implementation of SBDD in tuberculosis research resulted in the identification of many antimycobacterial drugs that were already subjected to clinical assessment, demonstrating their utility in the drug discovery and development framework. Although there is a requirement for more work to accelerate the identification of anti-TB medicines, the status of CADD in TB is a promising direction.

Another aspect of CADD is the nascent adoption of machine learning (ML) approaches in drug development research. With the increasing amount of data accessible and the sophisticated computers available, the world is witnessing a surge in drug discovery research incorporating artificial intelligence (AI). The use of ML has swept throughout the globe; integrating ML and deep learning (DL) strategies allows for effective and vast solutions to issues related to drug design. This significant change in the drug development framework facilitates interventions in personalized medicine and several relevant disorders, such as cancer. While there are currently numerous FDA-approved applications of AI in the healthcare sector holistically [251], the technology has not yet produced a viable medication candidate, but this may not be too far in the future.

Presently, AI research addressing tuberculosis (TB) is typically concerned with diagnoses and treatment results. The full adoption of AI will accelerate the discovery of innovative and effective anti-TB medicines. Eventually, AI might reduce the tremendous burden imposed by TB on the world’s health systems. Another critical area of CADD is the maturity of quantum computing and the perfection of clustered regularly interspaced short palindromic repeats (CRISPR) [252]. These will revolutionize personalized medicine and TB treatment in the future. Quantum computing can overcome most of the challenges with the current computational approaches and the ability to proffer exact solutions to the Schrödinger approximation equation for larger and more complex biomolecular systems. This procedure will be a great relief to humanity in the future, as the scourge of TB may be a thing of the past. CRISPR, a technique capable of gene modification, could be a viable tool for the future eradication of TB. It could help affect treatment of TB at the latency stage and possibly eliminate the bacterium via DNA modification. Hopefully, SBDD approaches to TB drug discovery and development in the future will introduce a paradigm shift with the hybrid incorporation of ML, AI, quantum computing, and CRISPR.

## Figures and Tables

**Table 1:** Comparison of the traditional method of drug development with CADD (computer-aided drug design).

The Traditional Method of Drug Development	CADD
It involves more trial-and-error processes	It is more logical
It involves blind screening	It is specific and mostly target-based
It is a more expensive approach to drug development	It minimizes the cost of drug development
It is a relatively more laborious and time-consuming approach	It reduces the duration required in the development of new drugs
It involves sequential steps	It entails steps that are not only sequential but are also parallel and straightforward.
It involves separate interdisciplinary drug development with more difficult processes	It coordinates interdisciplinary drug development with easier processes.

**Table 2:** New Mtb drugs and their mode of action in different clinical trial phases.

Drug	Class of Compound	Target	Approach	Clinical Trial Phase
Linezolid	Oxazolidinone	50S ribosomal subunit	Revisiting established targets (repurposing)	Phase 2
Sutezolid	Oxazolidinone	50S ribosomal subunit	Revisiting established targets (repurposing)	Phase 1
Bedaquiline (TMC207)	Diarylquinoline	ATP synthase	Phenotypic-HTS	Approved
TBAJ-587	Diaryquinoline	ATPsynthase	Revisiting novel target	Preclinical
Delamanid	Nitroimidazoles	Cell wall biosynthesis	HTS; modification of drug scaffold	Approved
Pretomanid	Nitroimidazoles	Cell wall biosynthesis	HTS; modification of drug scaffold	Approved
Telacebec (Q203)	Imidazopyridine amides	Cytochrome bc1 complex	HTS	Phase 2
Gatifloxacin	Quinolones	DNA gyrase; *gyrA*, *gyrB*	Revisiting established targets (repurposing)	Phase 3/4
Moxifloxacin	Quinolones	DNA gyrase; *gyrA*, *gyrB*	Revisiting established targets (repurposing)	Phase 3/4
Benzothiazinone (BTZ-043)	Benzothiazole	Decaprenylphosphoryl-β-_D_-ribose-2′-oxidase (DprE1)	HTS	Phase 2
Macozinone (PBTZ)	Benzothiazole	DprE1	HTS	Phase 2
OPC-167832	Carbostyril	DprE1	HTS	Phase 2
TBA7371	Azaindoles	DprE1	HTS; modification of drug scaffold	Phase 2A
Clofazimine	Riminophenazine	Electrogenic pathway, reduced by NADH dehydrogenase II	Revisiting established targets (repurposing)	Approved
SPR720	Benzimidazole class	GyrB ATPase	Revisiting established target (repurposing)	Phase 2
SQ109	Ethylenediamine	Inhibition of MmpL3, MenA, and MenG and ATP	HTS; modification of drug scaffold	Phase 2
GSK 070	Oxaborole	Leucine tRNA synthase	Revisiting established target (repurposing)	Phase 2
Delpazolid	Oxazolidinones	Ribosomal subunit	Revisiting established targets (repurposing)	Phase 2
OTB-658	Oxazolidinones	Ribosomal subunit	Revisiting established targets (repurposing)	Preclinical
TBI-223	Oxazolidinones	Ribosomal subunit	Revisiting established targets (repurposing)	Phase 1
Contezolid	Oxazolidinones	Ribosomal subunit	Modification of drug scaffold	Phase 3
Contezolid acefosamil (prodrug)	Oxazolidinones	Ribosomal subunit	Modification of drug scaffold	Phase 3
Sanfetrinem	Carbapenem	Cell wall biosynthesis	Revisiting established target	Phase 2
Sanfetrinem cilexetil (prodrug)	Carbapenem	Cell wall biosynthesis	Revisiting established target	Phase 2

Source: [70,71,72,73,74,75,76,77,78].

**Table 3:** Drug discovery by SBDD computational approaches.

Drug	Target	Target Disease	Computational Methods	Refs.
Epalrestat 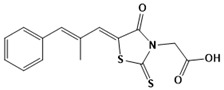	Aldose reductase	Diabetic neuropathy	MD and SBVS	[111]
Amprenavir 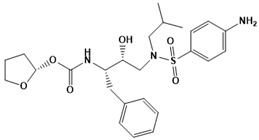	Antiretroviral protease	HIV	Protein modeling and molecular dynamics (MD)	[108,109]
Dorzolamide 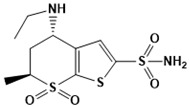	Carbonic anhydrase	Glaucoma, cystoid macular edema	Fragment-based screening	[112]
Flurbiprofen 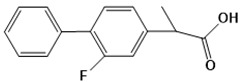	Cyclooxygenase-2	Rheumatoid arthritis, osteoarthritis	Molecular docking	[113,114]
Isoniazid 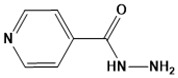	InhA	TB	SBVS and pharmacophore modeling	[115]
Pim-1 kinase inhibitors (E)-5-(4-hydroxybenzylidene)-2-iminothiazolidin-4-one 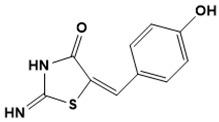 3-fluoro-4-((4-(isopropylamino)-5-nitropyrimidin-2-yl)amino)benzoic acid 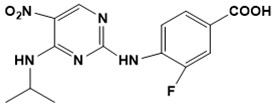 4-(benzofuran-2-yl)-6-ethyl-2H-chromen-2-one 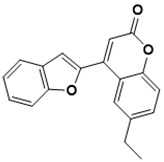	Pim-1 kinase	Cancer	Hierarchical multistage VS	[116]
STX-0119 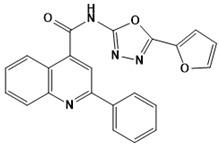	STAT3	Lymphoma	SBVS	[117]
Raltitrexed 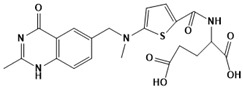	Thymidylate synthase	HIV	SBDD	[98]
Norfloxacin 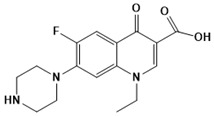	Topoisomerase II, IV	Urinary tract infection	SBVS	[118]
Cimetidine 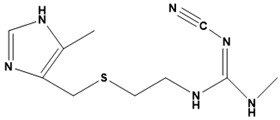	Histamine H2 receptor antagonist	Gastrointestinal disorder (ulcer)	SBVS	[119]
Zanamivir 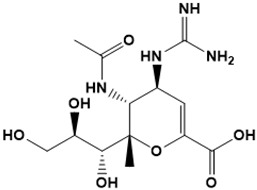	Neuraminidase inhibitor	Influenza	SBVS	[120]
Zolpidem 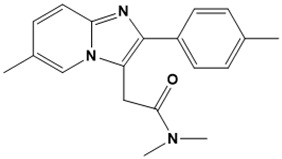	GABAA receptor agonist	Insomnia	SBVS	[121]
Imatinib 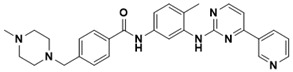	Bcr-Abi tyrosine-kinase inhibitor	Cancer	SBVS	[122]
Raltegravir 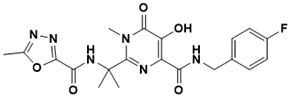	HIV integrase strand transfer inhibitor	HIV/AIDS	SBVS	[123]

**Table 4:** Successful SBVS approaches on anti-Mtb and activities of the best compounds *. A summary of Mtb pathways is available in the supporting information.

System	PDB Structures	Function	Anti-Mtb Activity	Ref.
L-alanine dehydrogenase	2VHW	Biosynthesis of l-alanine	IC50/35.5 μM ^b^	[127]
L-alanine dehydrogenase	4LMP	Biosynthesis of l-alanine	MIC/1.53 μM	[128]
L-alanine dehydrogenase	2VOJ	Biosynthesis of l-alanine	MIC/11.81 µM	[129]
7,8-diaminopelargonic acid synthase	3TFU	Biotin biosynthesis pathway	MIC/25 μM	[129]
7,8-diaminopelargonic acid synthase	3TFU	Biotin biosynthesis pathway	MIC/7.86 μM	[130]
Cyclopropane mycolic acid synthase 1	1KPH	Cell wall	MIC50/5.1 μM	[131]
l,d-transpeptidase 2	3TUR	Cell wall	MIC94/25.0 μM MIC89/0.2 μM	[132]
GlmU protein [58]	3ST8 ^a^	Cell wall	IC50/9.0 μM ^b^	
NAD⁺-dependent DNA ligase A	1ZAU/1TAE	DNA metabolism	MIC_50_/15 µM	[133]
Flavin-dependent thymidylate synthase	2AF6 ^a^	DNA metabolism	MIC90/125 μM	[134]
Flavin-dependent thymidylate synthase	2AF6	DNA metabolism	IC29/100 μM ^b^	[135]
DNA gyrase	4BAE	DNA topology	MIC/7.8 µM	[136]
Dihydrofolate reductase	Mtb: 1DF7; human: 1OHJ	Folate pathway	MIC/25 μM	[137]
Salicylate synthase	3VEH	Iron acquisition	MIC99/156 μM	[138]
Transcription factor IdeR	1U8R	Iron acquisition control	MIC90/17.5 μg/ml	[139]
Flavin-dependent oxidoreductase MelF	2WGK	Needed to withstand ROS-and RNS-induced stress	MIC/13.5 μM	[140]
Leucyl-tRNA synthetase	2V0C	Protein synthesis	MIC/25 µM	[141,142]
3-dehydroquinate dehydratase	2Y71	Shikimate pathway	MIC/6.25 µg/mL	[143]
3-dehydroquinate dehydratase	15 PDB structures	Shikimate pathway	MIC/100 mg/ml	[144]
Haloalkane dehalogenase	2QVB	Unknown	Kd/3.37 µM ^b^	[145]

* Structures are provided in Table 5. ^a^ Ligand-based approach and ^b^ in vitro enzymatic essays. PDB (Protein Data Bank).

**Table 5:** Structure of identified molecules with the best anti-Mtb activity or enzymatic inhibition.

Structure	IUPAC Name	Enzymatic Inhibition
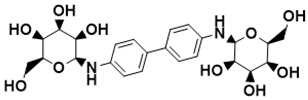	(2S,2′S,3S,3′S,4R,4′R,5R,5′R,6S,6′S)-6,6′-([1,1′-biphenyl]-4,4′-diylbis(azanediyl))bis(2-(hydroxymethyl)tetrahydro-2H-pyran-3,4,5-triol)	Biosynthesis of l-alanine [127]
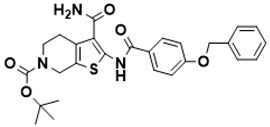	tert-butyl 2-(4-(benzyloxy)benzamido)-3-carbamoyl-4,7-dihydrothieno [2,3-c]pyridine-6(5H)-carboxylate	Biosynthesis of l-alanine [128]
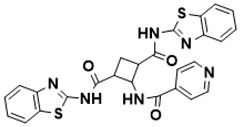	N^1^, N^3^-bis(benzo[d]thiazol-2-yl)-2-(isonicotinamido)cyclobutane-1,3-dicarboxamide	Biosynthesis of l-alanine [129]
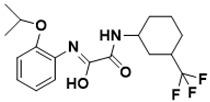	(*Z*)-*N*-(2-isopropoxyphenyl)-2-oxo-2-((3-(trifluoromethyl)cyclohexyl)amino)acetimidic acid	Biotin biosynthesis pathway [129]
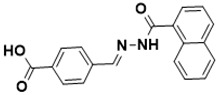	(*E*)-4-((2-(1-naphthoyl)hydrazono)methyl) benzoic acid	Biotin biosynthesis pathway [130]
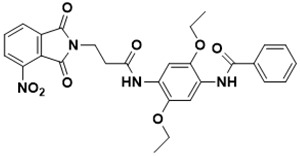	*N*-(2,5-diethoxy-4-(3-(4-nitro-1,3-dioxoisoindolin-2-yl)propanamido)phenyl) benzamide	Cell wall [131]
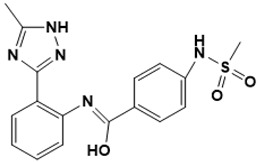	(*Z*)-*N*-(2-(5-methyl-1H-1,2,4-triazol-3-yl) phenyl)-4-(methylsulfonamido)benzimidic acid	Cell wall [132]
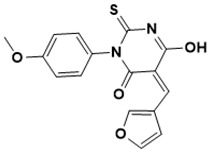	(*Z*)-5-(furan-3-ylmethylene)-6-hydroxy-3-(4-methoxyphenyl)-2-thioxo-2,5-dihydropyrimidin-4(3H)-one	Cell wall [133]
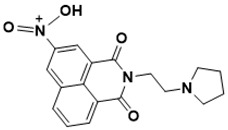	*N*-(1,3-dioxo-2-(2-(pyrrolidin-1-yl)ethyl)-2,3-dihydro-1H-benzo[*de*]isoquinolin-5-yl)-N-oxohydroxylammonium	DNA metabolism [134]
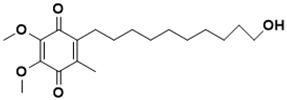	2-(10-hydroxydecyl)-5,6-dimethoxy-3-methylcyclohexa-2,5-diene-1,4-dione	DNA metabolism [135]
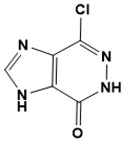	7-chloro-3,5-dihydro-4H-imidazo [4, 5-*d*]pyridazin-4-one	DNA metabolism [136]
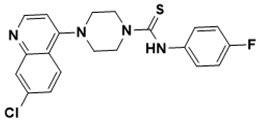	4-(7-chloroquinolin-4-yl)-*N*-(4-fluorophenyl)piperazine-1-carbothioamide	DNA topology [137]
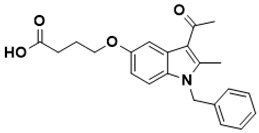	4-((3-acetyl-1-benzyl-2-methyl-1H-indol-5-yl)oxy)butanoic acid	Folate pathway [138]
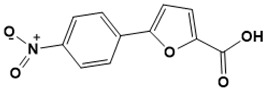	5-(4-nitrophenyl)furan-2-carboxylic acid	Iron acquisition [139]
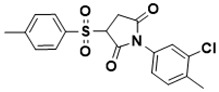	1-(3-chloro-4-methylphenyl)-3-tosylpyrrolidine-2,5-dione	Iron acquisition control [140]
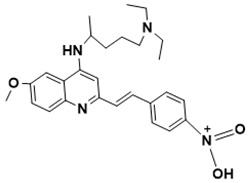	(E)-*N*-(4-(2-(4-((5-(diethylamino)pentan-2-yl)amino)-6-methoxyquinolin-2-yl)vinyl)phenyl)-N-oxohydroxylammonium	Needed to withstand ROS- and RNS-induced stress [141]
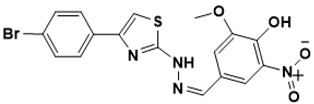	(Z)-4-((2-(4-(4-bromophenyl)thiazol-2-yl)hydrazono)methyl)-2-methoxy-6-nitrophenol	Protein synthesis [142]
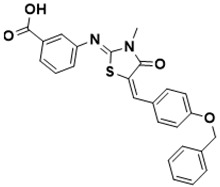	3-(((Z)-5-((E)-4-(benzyloxy)benzylidene)-3-methyl-4-oxothiazolidin-2-ylidene)amino)benzoic acid	Shikimate pathway [143]
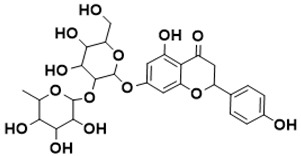	7-((4,5-dihydroxy-6-(hydroxymethyl)-3-((3,4,5-trihydroxy-6-methyltetrahydro-2H-pyran-2-yl)oxy)tetrahydro-2H-pyran-2-yl)oxy)-5-hydroxy-2-(4-hydroxyphenyl)chroman-4-one	Shikimate pathway [144]
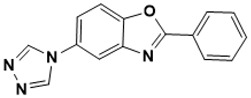	2-phenyl-5-(4H-1,2,4-triazol-4-yl)benzo[d]oxazole	Unknown [145]

**Table 6:** Accessible public and commercial repositories on TB drug development.

Database	Number of Compounds	Website *	Ref.
** Enamine REAL	700 million	https://enamine.net/	[155]
** ZINC	230 million	http://zinc.docking.org/	[156]
** GDB-17	166 billion	http://gdb.unibe.ch/	[157]
** PubChem	97 million	https://pubchem.ncbi.nlm.nih.gov/	[147]
** ChemSpider [142]	77 million	http://www.chemspider.com/	[158]
*** eMolecules	24.6 million	http://www.emolecules.com	
** ChEMBL	1.9 million	https://www.ebi.ac.uk/chembl/	[159]
*** ASINEX	600,000	http://www.asinex.com	
** NCI	460,000	https://cactus.nci.nih.gov/download/roadma/	[160]

Note: * links accessed 5 September 2021, ** and *** indicate public and commercial types of databases, respectively.

**Table 7:** Accessible websites to retrieve software for CADD.

Purpose	Program	Website *	Refs.
Prediction of binding sites and drugability	** fpocket	https://github.com/Discngine/fpocket	[161,162]
	** PockDrug	http://pockdrug.rpbs.univ-paris-diderot.fr/cgi-bin/index.py?page=home	[163]
	** PocketQuery	http://pocketquery.csb.pitt.edu/	[164]
	** PASS	http://www.ccl.net/cca/software/UNIX/pass/overview.html	[165]
Docking	** Autodock	http://autodock.scripps.edu/	[166]
	*** GOLD	https://www.ccdc.cam.ac.uk/solutions/csddiscovery/components/gold/	[167]
	*** Glide	https://www.schrodinger.com/glide/	[168]
	*** FlexX	https://www.biosolveit.de/flexx/index.html	[169]
QSAR	*** SeeSAR	https://www.biosolveit.de/SeeSAR/	[170]
	** Open3DQSAR	http://open3dqsar.sourceforge.net/?Home	[171]
	** ChemSAR	http://chemsar.scbdd.com/	[172]
ADMET	*** QikProp	https://www.schrodinger.com/qikprop	[173]
	*** ADMET Predictor	https://www.simulations-plus.com/software/overview/	[174]
	** admetSAR	http://lmmd.ecust.edu.cn/admetsar1/home/	[175,176,177]
	** VirtualToxLab	http://www.biograf.ch/index.php?id=home	[15,178,179,180]

Note: * links accessed 5 September 2021, ** and *** mean freely and commercially accessible, respectively.

**Table 8:** Studies involving SBVS molecular-docking approaches against Mtb enzymes.

Program	Library of Compounds Screened	Enzyme (Function)	Ref.
AutoDock Vina	FDA-approved: DrugBank (1932); eLEA3D (1852)	MurB and MurE (peptidoglycan biosynthesis)	[207]
	ChemDiv dataset (135,755)	DprE1 (arabinogalactan biosynthesis)	[208]
	NCI; Enamine; Asinex; ChemBridge; Vitas-M Lab (total: 5.6 million)	InhA (mycolic acid biosynthesis)	[209]
AutoDock 4.0	Super Natural II database (570)	RmlD (carbohydrate biosynthesis)	[210]
CDOCKER	Enamine REAL database (4.5 million)	BioA (biotin biosynthesis)	[203]
Frigate	ZINC database (2 million)	Antigen 85c (lipid metabolism)	[211]
Glide	FDA-approved (6282)	LipU (lipid hydrolysis)	[212]
	ChEMBL antimycobacterial (30,789)	DprE1 (arabinogalactan biosynthesis)	[213]
	FDA-approved (3176)	PknA (protein kinase)	[214]
	Preselected from Maybridge database (1026)	InhA (mycolic acid biosynthesis)	[215]
	Preselected from DrugBank database (1082)	AroB (shikimate pathway)	[216]
GOLD	Drugs Now subset of ZINC database (409, 201)	EthR (transcriptional regulator)	[205]
GOLD and Plants	Preselected from Enamine database (2050)	MbtI (mycobactin synthesis)	[138]
GOLD and RFScore	Selection from 9 million compounds (4379)	AroQ (Shikimate pathway)	[217]
UCSF Chimera	CDD-823953; GSK-735826A	PyrG and PanK (siosynthesis of DNA and RNA)	[192]

## Data Availability

The data used are freely accessible to the general public in the various links provided in different sections of this article with the date accessed.
